# Supplementary material for: Obesity and Dyslipidemia Synergistically Exacerbate Psoriatic Skin Inflammation
Source: Int J Mol Sci. 2022 Apr 13;23(8):4312. doi: 10.3390/ijms23084312 (PMC9032572; doi:10.3390/ijms23084312)
Supplement: Supplementary file 1 [file ijms-23-04312-s001.zip › Figure S1.pdf]

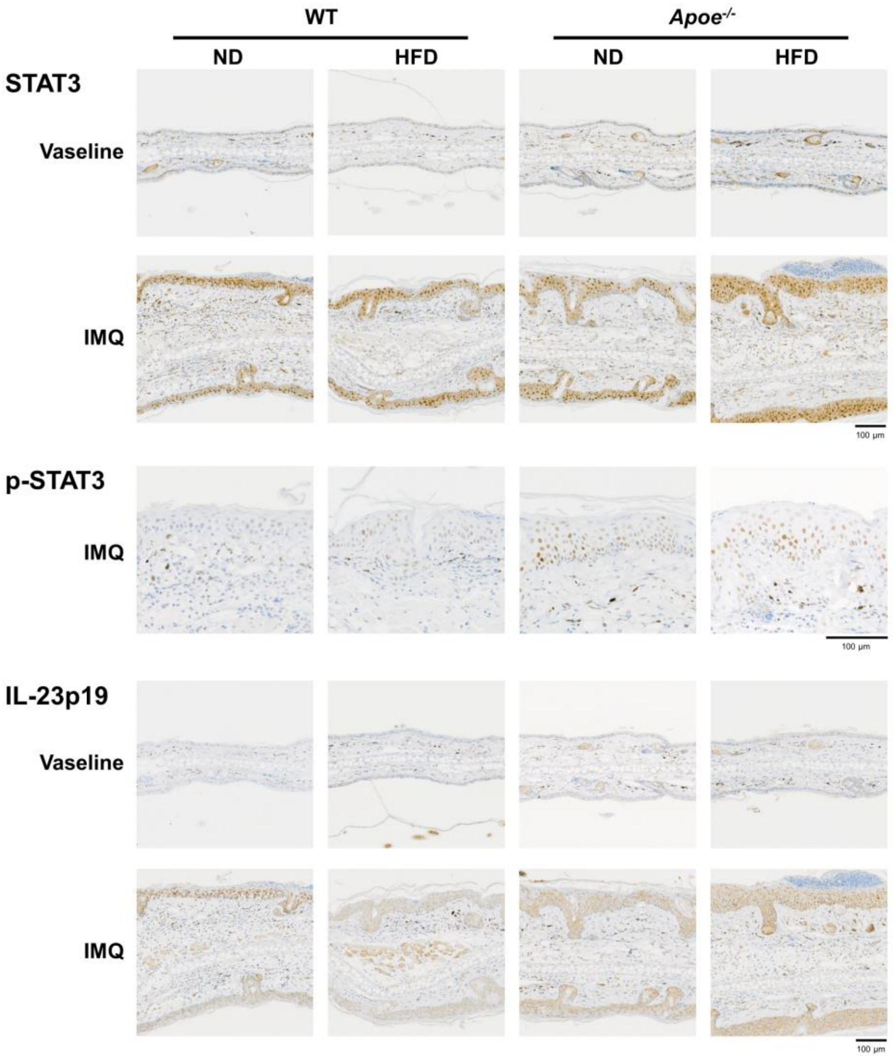

Figure S1. Immunohistochemical staining for signal transducer and activator of transcription (STAT)-3, phospho-STAT3, and IL-23. Wild-type (WT) and *Apoe*$^{-/-}$ mice were fed normal or high-fat diet for 10 weeks. Imiquimod (IMQ) cream (25 mg) or Vaseline (control) was topically applied to the ear for 5 consecutive days. Immunohistochemical staining for STAT3, phospho-STAT3, and IL-23p19 was performed on skin tissues. Images for STAT3 and IL-23p19 are presented at low magnification; images for phospho-STAT3 are presented at high magnification. Scale bars = 100 μm. IMQ, imiquimod; WT, wild-type; ND, normal diet; HFD, high-fat diet.
