# Supplementary material for: Obesity and Dyslipidemia Synergistically Exacerbate Psoriatic Skin Inflammation
Source: Int J Mol Sci. 2022 Apr 13;23(8):4312. doi: 10.3390/ijms23084312 (PMC9032572; doi:10.3390/ijms23084312)
Supplement: Supplementary file 1 [file ijms-23-04312-s001.zip › Figure S2.pdf]

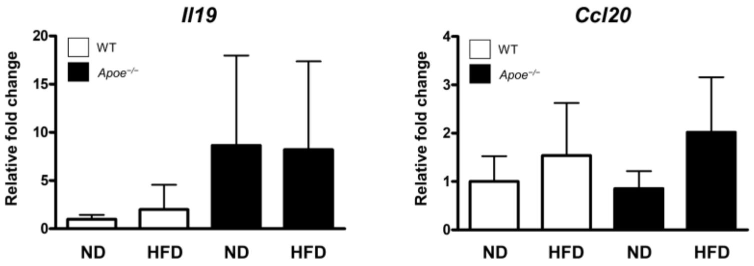

Figure S2. mRNA expression of *Il19* and *Ccl20* in skin tissues from obese and hyperlipidemic mice. RNA samples were extracted from the ear tissues of wild-type and *Apoe*$^{-/-}$ mice that were fed normal or high-fat diet, and gene expression levels were determined by real-time quantitative PCR. Data from the Vaseline-treated groups were extracted from the data presented in Figure 3. WT, wild-type; ND, normal diet; HFD, high-fat diet.
