# Supplementary material for: Obesity and Dyslipidemia Synergistically Exacerbate Psoriatic Skin Inflammation
Source: Int J Mol Sci. 2022 Apr 13;23(8):4312. doi: 10.3390/ijms23084312 (PMC9032572; doi:10.3390/ijms23084312)
Supplement: Supplementary file 1 [file ijms-23-04312-s001.zip › Figure S3.pdf]

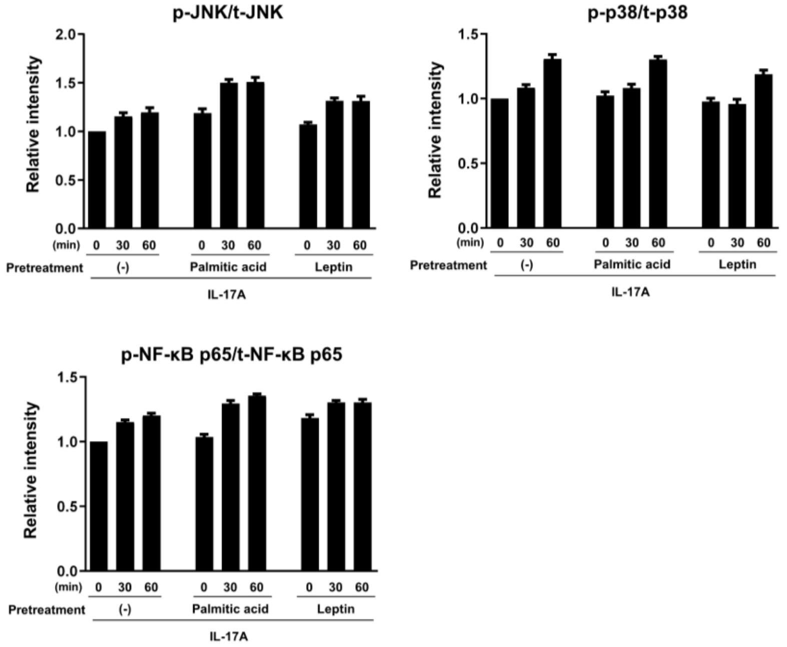

Figure S3. Effects of palmitic acid and leptin on the signaling pathways in epidermal keratinocytes. Quantification of phosphorylated proteins presented in Figure 6b. Neonatal human epidermal keratinocytes (NHEKs) were pretreated with palmitic acid (100 μM) or leptin (100 ng/ml) for 2 hours and then treated with IL-17A (30 ng/ml) for the indicated time. Phospho- or total-phosphorylation of c-Jun N-terminal kinase (JNK), p38, and NF-κB p65 were detected using specific antibodies. Intensities of bands of indicated proteins were quantified by ImageJ. The relative intensities of each phosphorylated protein were divided by those of total-JNK, p38, and NF-κB p65, respectively. Levels relative to non-stimulated cells were calculated.
