# Supplementary material for: Obesity and Dyslipidemia Synergistically Exacerbate Psoriatic Skin Inflammation
Source: Int J Mol Sci. 2022 Apr 13;23(8):4312. doi: 10.3390/ijms23084312 (PMC9032572; doi:10.3390/ijms23084312)
Supplement: Supplementary file 1 [file ijms-23-04312-s001.zip › Table S1.pdf]

Table S1. The list of primers used in this study.

Primer	Gene Accession Number	Sequence		Amplicon Size (bp)
		Forward (5→3)	Reverse (5→3)	
Gapdh	NM_008084.2	atcaagaaggtggtgaagca	gacaacctggtcctcagtgt	75
Il1b	NM_008361.3	agttgacggaccccaaaag	agctggatgctctcatcagg	75
Il6	NM_031168.1	aacgatgatgcacttgcaga	ccagaggaaattttcaataggc	113
Il17a	NM_010552.3	cagggagagcttcatctgtgt	gctgagctttgagggatgat	94
Il17c	AF458061.1	cctctagctggaacacagtgc	gcggttctcatctgtgtcg	114
Tnf	NM_013693.2	catcttctcaaaattcgagtgaca	tgggagtagacaaggtacaaccc	175
Il23a	NM_031252.2	caccagcgggacatatgaa	ccttgtgggtcacaaccat	100
Il22	NM_016971.2	tgacgaccagaacatccaga	aatcgccttgatctctccac	85
Il19	NM_001009940.1	tggagaacctcaggagcatt	gaatgtcagcaggttgttgg	65
Cxcl1	NM_008176.3	cttgaccctgaagctccctt	gttgtcagaagccagcgttc	127
Cxcl3	NM_203320.3	ccccaggcttcagataatca	tctgatttagaatgcaggtcctt	110
Cxcl5	NM_009141.3	tagagccccaatctccacac	ggagctggaggctcattgt	88
Ccl20	NM_016960.2	aactgggtgaaaagggctgt	gtccaattccatcccaaaaa	86
Defb4	NM_019728.4	cagtcatgaggatccattacctt	aatttgggtaaaggctgcaa	77
S100a8	NM_013650.2	gtgtcctcagtttgtgcagaat	tgagatgccacacccacttt	126
S100a9	NM_001281852.1	gacaccctgacaccctgag	tgagggcttcatttctcttctc	96
Krt16	NM_001313958.1	atgagctgaccctgtccaga	ctcaaggcaagcatctcctc	106
GAPDH	NM_002046.5	agccacatcgctcagacac	aatacgaccaaatccgttgact	62
CCL20	NM_004591.2	gtgctgctactccacctctg	gcattgatgtcacagccttca	143
CXCL1	NM_001511.3	catcgaaaagatgctgaacagt	ataagggcagggcctcct	94
CXCL8	NM_000584.3	cagagacagcagagcacaca	gcactccttggcaaaactgc	167
IL1B	NM_000576.2	aaagcttggtgatgtctggtc	ggacatggagaacaccacttg	89
TNF	NM_000594.3	agcccatgttgtagcaaacc	tctcagctccacgccatt	94
IL19	NM_153758.2	cagagtcatccatgacaactatgat	cgagctctcccagggatt	71
IL17RA	NM_014339.6	catcctgctcatcgtctgc	gccatcggtgtatttggtgt	85
IL17RC	NM_153460.3	ttgaaccacacagacctggtt	tcctaacggagtcaggttcc	70
TNFRSF1A	NM_001065.3	cacaagccacagagcctaga	gaattccttccagcgcaac	87
LEPR	NM_002303.5	aaaaatgtcactttactttggaagc	tttcccacatcttctgacca	119
